# Some Methods of Teaching Elementary Operative Dentistry

**Published:** 1904-06

**Authors:** William H. Potter

**Affiliations:** Boston, Mass.


### SOME METHODS OF TEACHING ELEMENTARY OPERATIVE DENTISTRY.[1]

BY WILLIAM H. POTTER, D.M.D., BOSTON, MASS.[2]

At the present day much is said as to the most approved methods of imparting information. It is generally conceded that the highest degree of success cannot be attained by the average individual unless the method employed is of the most approved sort. The methods of dental education have advanced coincidentally with those of other branches of education. If we go back twenty years we will find that the way by which a man was taught the elements of operative dentistry was to allow him to operate upon a patient. The mouth of the patient was his work-bench and his subject for operation. He sat down or stood up to it and began to learn the nature of his instruments. He tried them in the mouth to see how they fitted different situations there to be found. He thus became acquainted with his instruments. He found that certain of them would not cut tooth-substance, and that certain of them would. He discovered the necessity for curves and angles. He found out the methods of applying force in the mouth, and so controlling it that it would go no farther than desired. This last piece of information was usually obtained through great hardship to the patient, as was evidenced by the laceration of parts surrounding the teeth due to the slipping of instruments. To be sure, these elementary things about operative dentistry were not learned from the patient with absolutely no preliminary training. It was the custom to set up teeth in plaster and work upon them before going into the mouth. But the operations upon teeth so set up were rather unsatisfactory, owing to the difficulty of mounting them in such a way as to imitate the conditions found in the mouth. Hence it was that students were very quickly put upon actual cases and allowed to acquire by the aid and forbearance of the patient the elements of operative work. This method was a decided injustice to the patient, and was undesirable for the

---

[1] Read before the American Academy of Dental Science, Boston, Mass., April 6, 1904.

[2] Assistant Professor of Operative Dentistry, Harvard Dental School.

student, inasmuch as it surrounded him with too many difficulties all at one time.

Modern methods of teaching operative dentistry insist, first of all, upon a knowledge of dental anatomy. This anatomy implies more than is likely to be learned from the course in general anatomy. It has to do with the external contour of the teeth, the peculiarities which distinguish one tooth from another, the number and location of cusps and fissures, the size and shape of the pulp-cavity, the proper occlusion of the teeth. Without the knowledge gained by such study it is inevitable that the beginner will make many false steps; as, for instance, the forming of undercuts in such a manner as to expose the pulp; the failure to find and properly treat all the root-canals; the improper use of drills in root-canals whereby false passages are made from the root-canal to the socket of the tooth. As a general surgeon who proposes to cut into the tissues of the arm should know in advance what muscles and vessels he will encounter, so the dental operator should first be sure of his anatomy before venturing to do the simplest work in the mouth. This seems a self-evident proposition, and yet only in comparatively recent time have we given sufficient importance to it. After the student has acquired dental anatomy, the modern method puts him at work upon teeth that are so arranged as to simulate the conditions found in the mouth. Great improvements have been made since the time when two or three teeth were set up in plaster on which the student began his work in operative dentistry.

I wish here to describe an apparatus which I came across in Vienna four years ago and which is now used at the Harvard Dental School. This apparatus is called in Vienna a "Phantom," and it came into existence in this way: It had been the custom in the Dental Department of the University of Vienna to use a human skull provided with full dentures for the demonstration of operations upon the teeth and for the first work of students in operative dentistry. This skull was mounted upon a standard and brought into such a position that it could be readily operated upon. In Vienna it is a comparatively easy matter to obtain a skull with a good set of teeth, and the expense of a skull is not great. Such a skull offered an excellent means for teaching students the methods of operating upon the teeth, and also gave them useful practice. If skulls were plenty enough, no better method could be devised than to use them in the teaching of the rudiments of operative dentistry. But even in Vienna, so bountiful in its supply of anatomical material, suitable skulls and teeth might not always be available, and it occurred to Messrs. Weiss & Schwartz, of Vienna, to make the apparatus which I have alluded to as a "Phantom," and which takes the place of the skull. The "Phantom" consists of two iron jaws bolted together so as to imitate the positions of the superior and inferior maxillæ. The two jaws are also attached to a post which can be clamped to a dental chair in place of the usual head rest. Thus fixed, it occupies the same relative position which a human head would occupy when undergoing a dental operation. The iron jaws are so arranged that extracted teeth can be inserted and fixed by means of plaster of Paris. Here, then, we have a simple apparatus which when filled with teeth simulates in the most important particulars the conditions found in the human mouth. On it the student can begin his operative experiences; he can get used to his instruments and learn how to apply force in the mouth. While the "Phantom" imitates the conditions of the mouth, it is undoubtedly easier to operate upon it than upon the mouth. The saliva is absent, the resisting lips and cheeks are absent, and, of no small importance, the nerves of the patient are absent. Thus for the beginning student some of the difficulties are removed and he is able to master the elementary things and be prepared for the more difficult. It is the custom at the Harvard Dental School to require each student to perform the more common operations upon the "Phantom" before they are performed in the mouth.

I am aware that there are sundry devices now made which are calculated to perform the same service as the "Phantom." There are rubber jaws in which teeth can be inserted and operated upon, and which are useful as far as they go. And then there are very complicated and expensive manikin heads, in which teeth can be placed. They are very useful, but are so expensive that they cannot well be furnished for each student. Composition teeth of celluloid and rubber have been made for operative purposes, but there is always the objection to artificial teeth that the student is cutting a substance which is essentially different from dentine or enamel.

A final point which I wish to touch upon is the method of teaching cavity preparations by means of plaster teeth of a size of perhaps ten times that of natural teeth. Such teeth, so far as I know, cannot be bought and must be manufactured at one's own laboratory. Here is a bicuspid form which I have used during the past year. I carved the original one myself, and then reproduced it by means of the fusible metal moulds which I show. It is rather a rough affair, but it serves accurately to express the teacher's idea as to how a cavity should be prepared. It is my custom to ask students to prepare cavities in these models and bring them to the class for criticism. It is thus possible to know with certainty whether a student has understood what has been said to him.

